# Current status of recombinant duck enteritis virus vector vaccine research

**DOI:** 10.3389/fvets.2025.1453150

**Published:** 2025-02-05

**Authors:** Wen-Feng Jia, An-Ping Wang, Zhi Wu, Xin-Nuo Lei, Yu-Ting Cheng, Shan-Yuan Zhu

**Affiliations:** Jiangsu Key Laboratory for High-Tech Research and Development of Veterinary Biopharmaceuticals, Jiangsu Agri-Animal Husbandry Vocational College, Taizhou, China

**Keywords:** duck enteritis virus, recombinant vector vaccine, genetic modification, waterfowl infectious disease, immune protection efficacy, avian vaccines

## Abstract

Duck enteritis virus (DEV), the pathogen of duck viral enteritis, belongs to the *α*-herpesvirus subfamily. Like other herpesviruses, it has a large genome with multiple non-coding and non-essential regions for viral replication. It is suitable as a live virus vector for inserting and expressing antigenic genes from other pathogens to develop multivalent vaccines. With the advancement of molecular biology research and experimental technology, genetic modification of the DEV genome has matured, leading to the successful construction of recombinant DEV live vector vaccines. These vaccines have demonstrated the ability to resist DEV and other pathogens, showing potential as recombinant viral vaccine vectors and playing a crucial role in the development of new avian vaccines. This article provides an overview of the progress of research on recombinant vaccines using DEV as the vector. It includes the biological characteristics of DEV and its advantages and limitations as a vaccine vector, methods for constructing recombinant DEV, the technical platform for efficiently building recombinant DEV, factors affecting the immune protection efficacy of recombinant DEV, and the application of recombinant DEV in vaccine development. Aiming to provide a reference for the development of duck enteritis virus vector-based vaccines.

## 1 Introduction

Vaccination and biosecurity stand as the foremost strategies for preventing and controlling infectious diseases, with vaccine immunization serving as a cornerstone in this endeavor. Although widely employed, live and inactivated vaccines come with inherent limitations, including the potential for virulence reversion in live vaccines, shorter immunization duration, and high production costs for inactivated vaccines. Poultry farming contends with a multifaceted infectious disease landscape, necessitating the prevention and control of a diverse array of diseases. This reality leads to challenges such as cumbersome vaccination procedures, high transportation costs for vaccines, contraindications between different vaccines, and stress-induced production losses in eggs or meat. Recent advancements in molecular biology technology have paved the way for the development of genetically engineered vaccines, offering a potential alternative to traditional vaccines. Genetically engineered vaccines encompass a variety of types, including subunit vaccines, vector vaccines, nucleic acid vaccines, gene-deficient live vaccines, and protein-engineered vaccines, among others. Among them, recombinant viral vector vaccine is a new type of vaccine that uses live virus as a carrier to carry and express antigenic genes of other pathogens. The exogenous genes integrated into the genome of the vector virus can continuously deliver exogenous antigenic proteins with the proliferation of the vector virus in cells, thereby inducing an immune response in the body.

Live viral vector vaccines have been shown to be effective in controlling infectious diseases, and a number of recombinant vector vaccines have been licensed, such as VECTORMUNE® HVT ND and VECTORMUNE® HVT AI from Ceva Animal Health Corporation, and VAXXITEK®HVT + IBD + ND, VAXXITEK®HVT + IBD, NEWXXITEK™HVT + ND from Merial-Boehringer Ingelheim, and so on (1, 2). By incorporating antigenic genes from multiple pathogens into viral vectors, a multi-plex polyvalent live vector vaccine can be constructed. Such a vaccine has the potential to prevent several infectious diseases with a single immunization, thereby reducing vaccine costs, simplifying the immunization process, and alleviating immunization pressures. Consequently, utilizing live vaccine vectors for expressing antigenicity genes of multiple pathogens represents a crucial direction for future vaccine research and development. Viral vectors suitable for live-vector vaccines include poxviruses (3–5), adenoviruses (6, 7), retroviruses (8), and herpesviruses (9–11), among which herpesviruses have become one of the popular vectors due to their infectiousness, large genome, and mature gene manipulation technology (12). This paper reviews the research progress of recombinant viral live-vector vaccines utilizing DEV as a vector, aiming to offer insights into the development of duck enteritis virus vector vaccines.

## 2 Biological characteristics of duck enteritis virus

**Figure 1:**
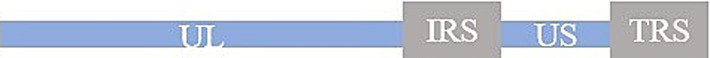
Schematic diagram of the genome structure of DEV.

Duck enteritis virus (DEV), also known as duck plague virus (DPV), is a significant pathogen that poses a serious threat to the waterfowl industry, which mainly infects birds of the order *Anseriformes*, causing duck viral enteritis (DVE) and induces acute, febrile, and septic infectious diseases (13). DEV belongs to the genus *Mardivirus*, family *Herpesviridae*, and subfamily Alpha-*herpesvirinae* (13). Its genome consists of double-stranded DNA with a molecular weight of about 160 kilobases (kb), including a long unique region (UL), a short unique region (US), an internal repetitive sequence (IRS), and a terminal repetitive sequence (TRS) in the arrangement UL-IRS-US-TRS (14), as shown in Figure 1. Being enveloped, the virus is sensitive to ether and chloroform.

The DEV genome consists of 67 open reading frames (ORFs) encoding non-structural and structural proteins, of which the non-structural proteins are mainly involved in regulating the processes of viral genome replication, transcription, and assembly, while the structural proteins mainly include capsid proteins, cortical proteins and envelope glycoproteins, which are involved in constituting and assembling the viral capsid, cortex and envelope, as well as in regulating the basic biological functions of the virus. Among them, envelope glycoproteins mainly include gB (UL27), gC (UL44), gD (US6), gE (US8), gG (US4), gH (UL22), gI (US7), gK (UL53), gL (UL1), which are the main genes affecting the basic biological functions of DEV such as DEV entry into the host cell, viral transmission, viral replication, viral assembly, and viral virulence (14). In addition to the envelope glycoprotein gene, there were significant sequence differences between strong and weak strains of DEV in the UL2, UL12, UL41, UL47, and US10 genes, suggesting that these five genes may be related to viral virulence (15). UL41, US3, UL28, UL53, UL24, and UL48 of DEV can block IFN-*β* activation and inhibit host innate immune response, which is thought to be associated with DEV immune escape (16–18).

DEV replicates primarily in the epithelial cells of the gastrointestinal mucosa and then spreads to the bursa, thymus, spleen and liver, where it replicates abundantly in epithelial cells and lymphoid cells. DEV levels in systemic tissues and organs correlate strongly with disease progression (19). DEV infection causes apoptosis and necrosis of lymphoid tissues, leading to immunosuppression and an increased risk of secondary bacterial infections. DEV also has a strong preference for vascular endothelial cells, and its replication in vascular endothelial cells of small blood vessels, small veins and capillaries leads to their destruction, which in turn causes severe hemorrhage, inflammatory responses and progressive degeneration of parenchymal organs. In addition, DEV can establish a latent infection in the trigeminal ganglia, which can be reactivated under the right conditions, leading to viral shedding and transmission (20). This reactivation may be due to immunosuppression in flocks due to stress from any cause. Latency and reactivation of DEV are significant factors contributing to outbreaks within both domestic and migratory waterfowl populations (13).

## 3 Construction of recombinant DEV

Constructing a recombinant DEV vector vaccine poses two primary challenges: first, inserting the target gene into the viral vector genome and stabilizing its presence; second, ensuring that the inserted foreign gene can be expressed normally. The advancements in molecular biology and gene editing technologies have facilitated the manipulation of the duck enteritis virus genome, including gene deletion and insertion, to a mature level. Currently, there are four main methods for constructing recombinant duck enteritis virus as follows.

### 3.1 Intracellular homologous recombination (HR)

**Figure 2:**
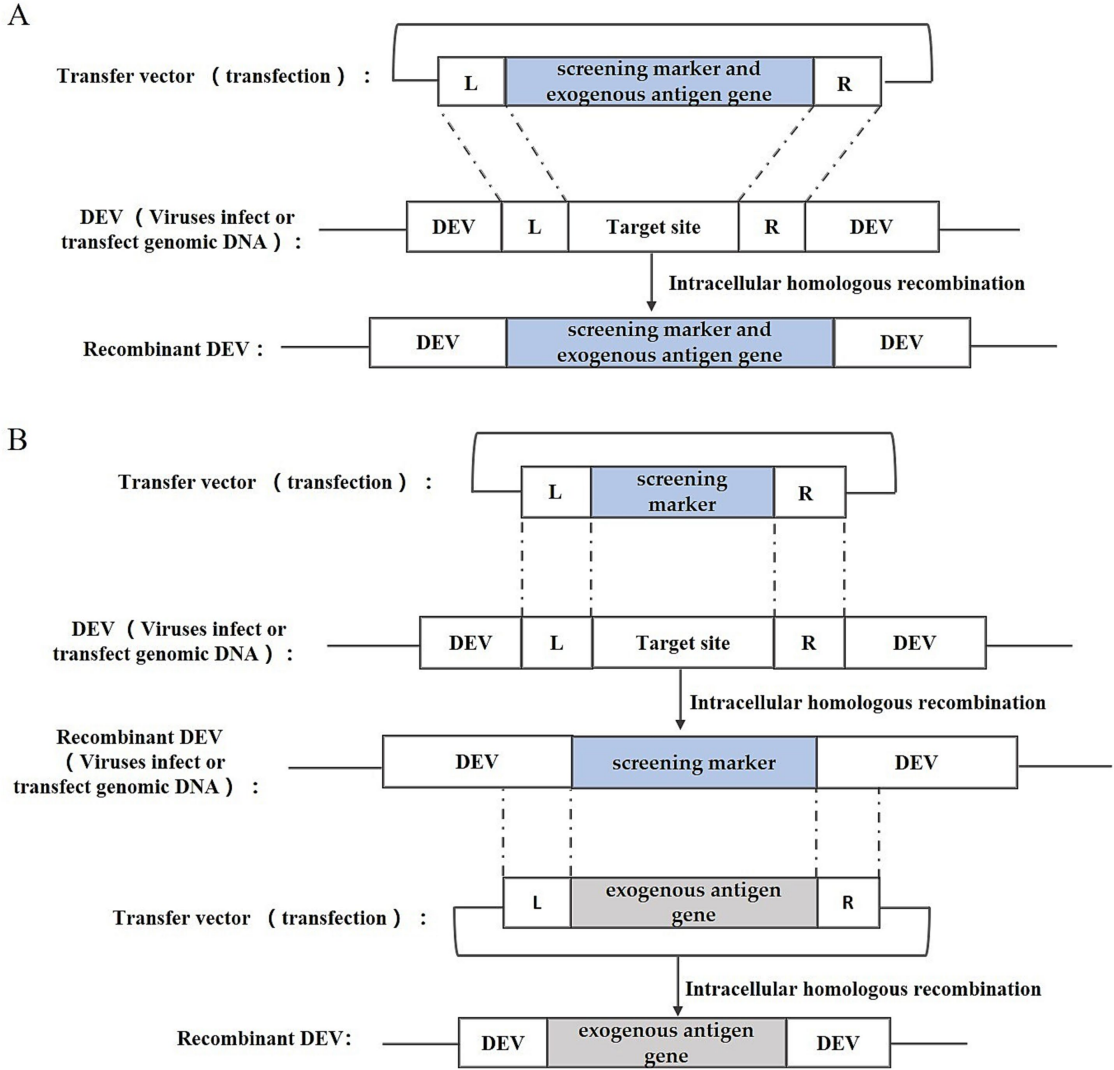
Recombinant DEV construction strategy based on intracellular homologous recombination. **(A)** One-step method; **(B)** Two-step method; L and R represent the left and right homologous arm sequences, respectively.

Intracellular homologous recombination represents a traditional approach for the generation of recombinant DEV. This method involves the insertion of an exogenous gene expression cassette into a specific region of the viral genome by utilizing a transfer vector equipped with homologous sequences flanking the insertion site. Subsequently, the repair and integration of the genome occur through the host cell’s homologous recombination mechanism. In addition to the exogenous gene expression cassette, the transfer vector typically incorporates a screening marker, such as the *Escherichia coli* (*E. coli*) guanine phosphoribosyl transferase (gpt) gene, *β*-galactosidase (LacZ) gene, or enhanced green fluorescent protein (EGFP) gene. These markers facilitate the subsequent screening and purification stages of the recombinant DEV. The construction strategy is illustrated in Figure 2, including one-step and two-step methods. One-step method: insert the exogenous gene into the DEV genome together with the screening marker to obtain the recombinant DEV; two-step method: insert the screening marker into the DEV genome first, and then replace the screening marker with the exogenous gene to obtain the recombinant DEV that expresses only the exogenous gene.

Liu et al. (21) utilized this method to fuse the *E. coli* gpt gene with the haemagglutinin (HA) gene of the highly pathogenic avian influenza virus (HPAIV) H5N1, and by linking it with the US2 gene and the gIgE gene of DEV, successfully integrated the gpt gene and the HA gene of H5N1 HPAIV into the DEV genome, thereby constructing two strains of rDEVs, which underwent subsequent screening and purification via the MPA/xanthine/hypoxanthine screening medium. Liu et al. (22) employed this method to insert the N, S, or S1 genes of infectious bronchitis virus (IBV) into the US10 locus of the DEV genome, initially recombining the EGFP reporter gene into DEV’s US10 gene and purifying the resultant rDEV-EGFP through screening for green fluorescent plaques, followed by crafting transfer vectors harboring IBV’s N, S, or S1 genes, and finally substituting the EGFP gene with IBV antigenic genes through a second round of homologous recombination, selecting the desired recombinant viruses based on fluorescence. Sun et al. (23) inserted the HA gene of the avian influenza virus H9N2 into the UL2 region of DEV by the same construction strategy.

This method is straightforward, involving only the creation of a transfer plasmid for recombinant virus recovery, and was widely adopted in its early stages. However, this approach has significant drawbacks. The likelihood of natural recombination in cells is very low, making it difficult to produce recombinant viruses.

### 3.2 Bacterial artificial chromosome (BAC) technology

**Figure 3:**
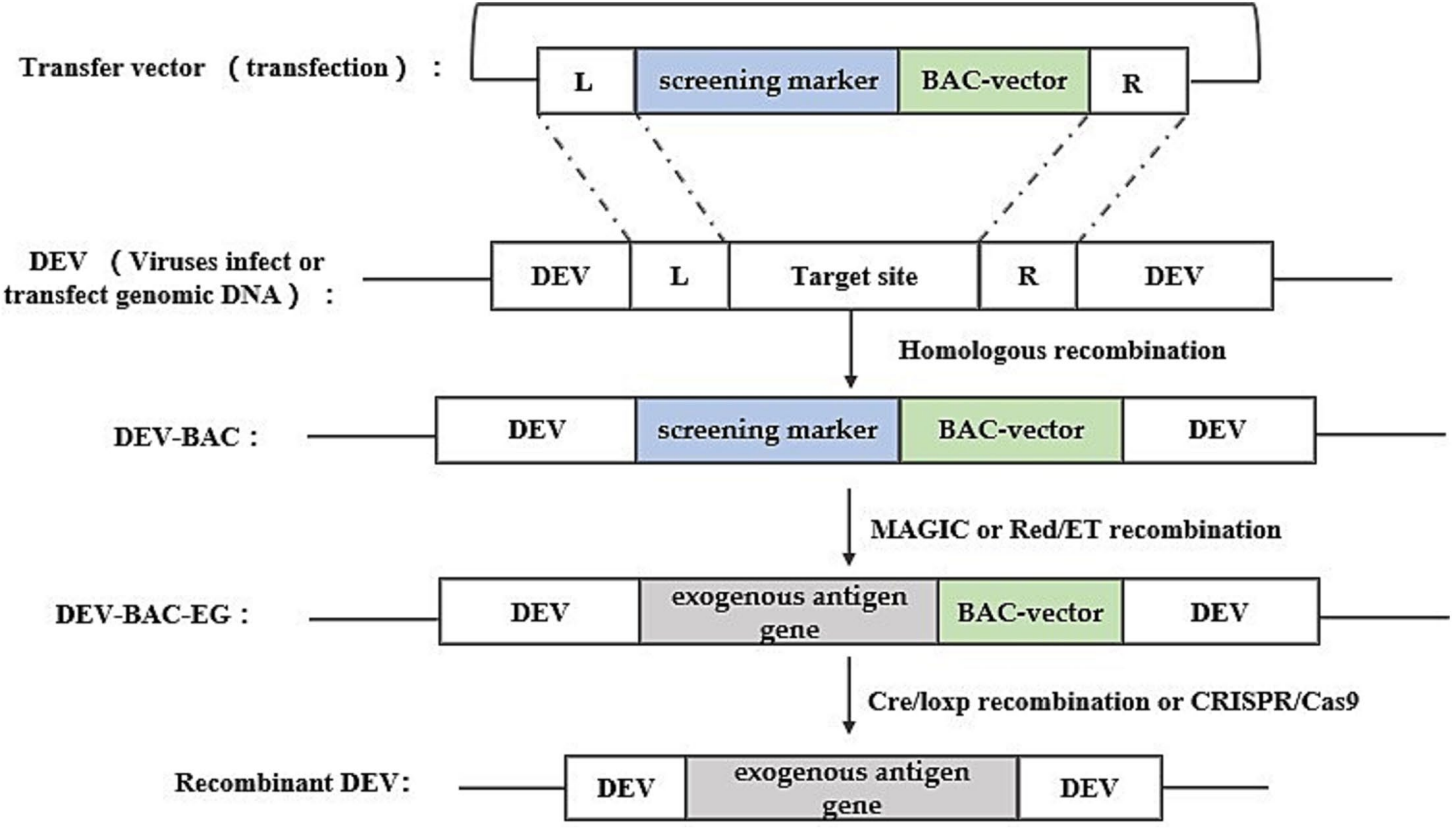
Recombinant DEV construction strategy based on bacterial artificial chromosome technology. Obtaining DEV-BAC as well as recombinant DEV was performed in avian cells, while obtaining DEV-BAC-EG was performed in *E. coli.* L and R represent the left and right homologous arm sequences, respectively.

Bacterial artificial chromosome technology is an efficient tool for genetic manipulation, particularly beneficial for studying viruses with large genomes like DEV. BAC, a low-copy plasmid based on the *E. coli* F-factor, is known for its large capacity, genetic stability, and straightforward manipulation. The inclusion of bacterial antibiotic resistance genes on the BAC vector aids in screening for *E. coli* containing the BAC-virus genome. Once DEV-BAC is successfully constructed, the DEV genome can be replicated in *E. coli* as a plasmid, manipulated using established Red homologous recombination techniques, and recombinant DEV can be rescued by transfecting BAC-DEV into host cells. The construction strategy is depicted in Figure 3. Initially, a transfer vector was constructed, incorporating the screening marker, BAC plasmid, and homologous arm sequences flanking the insertion site. Subsequently, DEV-BAC was generated through homologous recombination. The exogenous gene (EG) was then introduced into DEV-BAC using either Red/ET recombineering or mating-assisted genetically integrated cloning (MAGIC) in *E. coli*, yielding DEV-BAC-EG. Finally, the BAC sequence was excised using Cre/LoxP recombination or CRISPR/Cas9.

Liu et al. (24) used this method with a two-step RedE/T recombination approach to insert seven variations of the duck tembusu virus (DTMUV) E gene into the US7/US8 intergenic region of the DEV genome, creating seven distinct pDEV-E BAC clones, which were subsequently utilized to generate the recombinant virus rDEV-E in chicken embryonic fibroblasts (CEFs) through a calcium phosphate precipitation approach. Similarly, Wang et al. (25) inserted the HA gene of H5N1 AIV into a non-coding region of the DEV genome between UL55 and LORF11.

BAC is a widely used platform for herpesvirus genetic manipulation, with numerous successful applications in developing gene deletion vaccines and recombinant live viral vector vaccines, particularly for herpesviruses like MDV and PRV (26, 27). Although BAC offers higher efficiency than traditional homologous recombination, creating recombinant BAC constructs remains labor-intensive and time-consuming.

### 3.3 Fosmid multi-fragment rescue system

**Figure 4:**
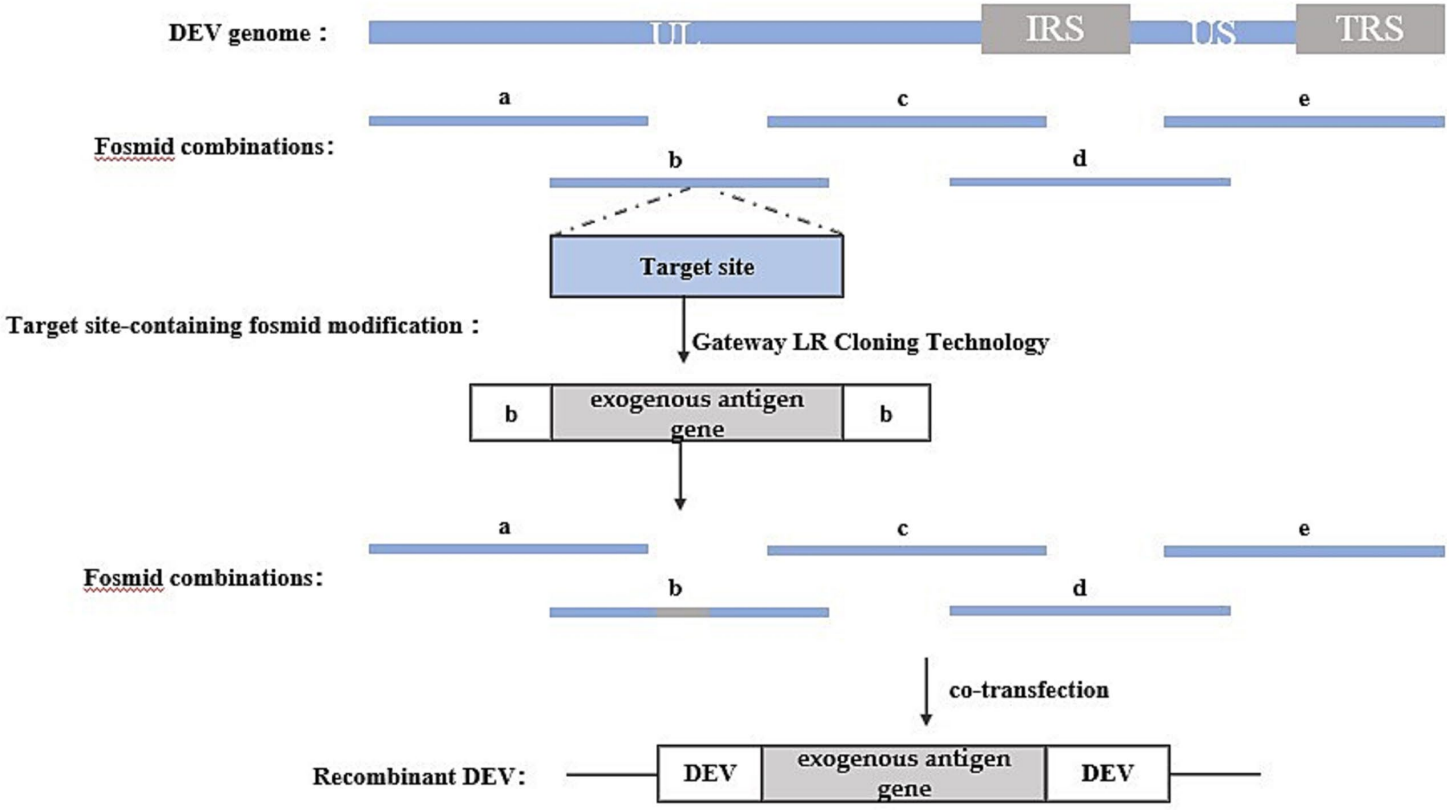
Recombinant DEV construction strategy based on Fosmid multi-fragment rescue system. Segments a, b, c, d, and e represent combinations of Fosmid plasmids that rescue infectious recombinant DEVs.

The Fosmid multi-fragment rescue system represents a relatively novel approach for constructing recombinant duck enteritis virus. Its fundamental principle involves the random fragmentation of the DEV genome into multiple segments with overlapping ends through physical or enzymatic cleavage. These fragments are subsequently cloned into Fosmid vectors. Different combinations of Fosmid plasmid DNAs covering the entire DEV genome are then co-transfected into host cells after enzymatic linearization, aiming to identify Fosmid plasmid combinations capable of rescuing infectious recombinant DEV, thus completing the construction of the reverse genetic platform. Exogenous genes can be inserted into specific sites within the Fosmid plasmid using conventional genetic engineering techniques. Subsequently, the Fosmid plasmid containing the exogenous gene and other plasmids in the combination are co-transfected into host cells after enzymatic linearization, and the success of the recombinant viruses can be assessed through the observation of cell lesions. The construction strategy is illustrated in Figure 4.

For instance, Zhao, et al. (28) utilized this method to insert the HA gene of H5 and H7 AIV into various loci of the DEV genome, such as UL41, US7/US8, SORF3/US2, US7/US8, and US8/US1. Similarly, Yang et al. (29) employed this method to insert the P1 and 3C genes of serotype 3 duck hepatitis A virus (DHAV-3) into UL26/UL27 and US7/8 loci.

One advantage of this method is that the rescued recombinant virus does not require purification, and recombinant viruses carrying exogenous genes can be obtained directly. However, a notable drawback lies in the necessity to construct numerous Fosmid vector plasmids to establish the reverse genetic manipulation platform. Additionally, screening for plasmid combinations with high recombination efficiencies is required. Failure to identify such combinations can lead to low intracellular homologous recombination efficiency, resulting in difficulties in rescuing recombinant viruses.

### 3.4 CRISPR/Cas9 gene editing technology

CRISPR/Cas9 gene editing technology, developed in recent years, represents a powerful tool for directly editing the genomes of herpes viruses. With its straightforward operation and high efficiency, this technology has found successful applications across various herpes viruses. The CRISPR/Cas9 gene editing system stems from an acquired immune system initially discovered in certain bacteria and archaea. Leveraging its working principle, this system has been harnessed for gene editing purposes. The Cas9 protein within the system exhibits nuclease activity, capable of binding to guide RNA (gRNA) and navigating to the target site under gRNA guidance. Upon reaching the target site, Cas9 cleaves the DNA, inducing a double-strand break (DSB) (30, 31), and the cleavage and breakage sites are generally in the PAM (Protospacer Adjacent Motif). The cut and break site is usually between the first three and four bases of the PAM (Protospacer Adjacent Motif) sequence, and the cell will activate the repair system to repair the break after the DSB is generated. The DSB repair system in eukaryotic cells consists of non-homologous end joining (NHEJ) and homology-directed repair (HDR) (32).NHEJ repair occurs at all stages of the cell life cycle, and can cause random base insertions and deletions, resulting in target gene shift mutations and the inability to encode the corresponding proteins, thus realizing the silencing and knockdown of target genes; In contrast, HDR repair predominantly occurs during the G2 and S phases of the cell cycle. It facilitates precise repair of DNA double-strand breaks by utilizing another homologous chromosome as a template. This repair mechanism can be utilized in cell lines and eukaryotic organisms with artificial templates, enabling targeted gene knockout, phenotype modification, and introduction of exogenous or reporter genes. This process represents a crucial mechanism for repairing DNA double-strand damage through homologous recombination (33).

**Figure 5:**
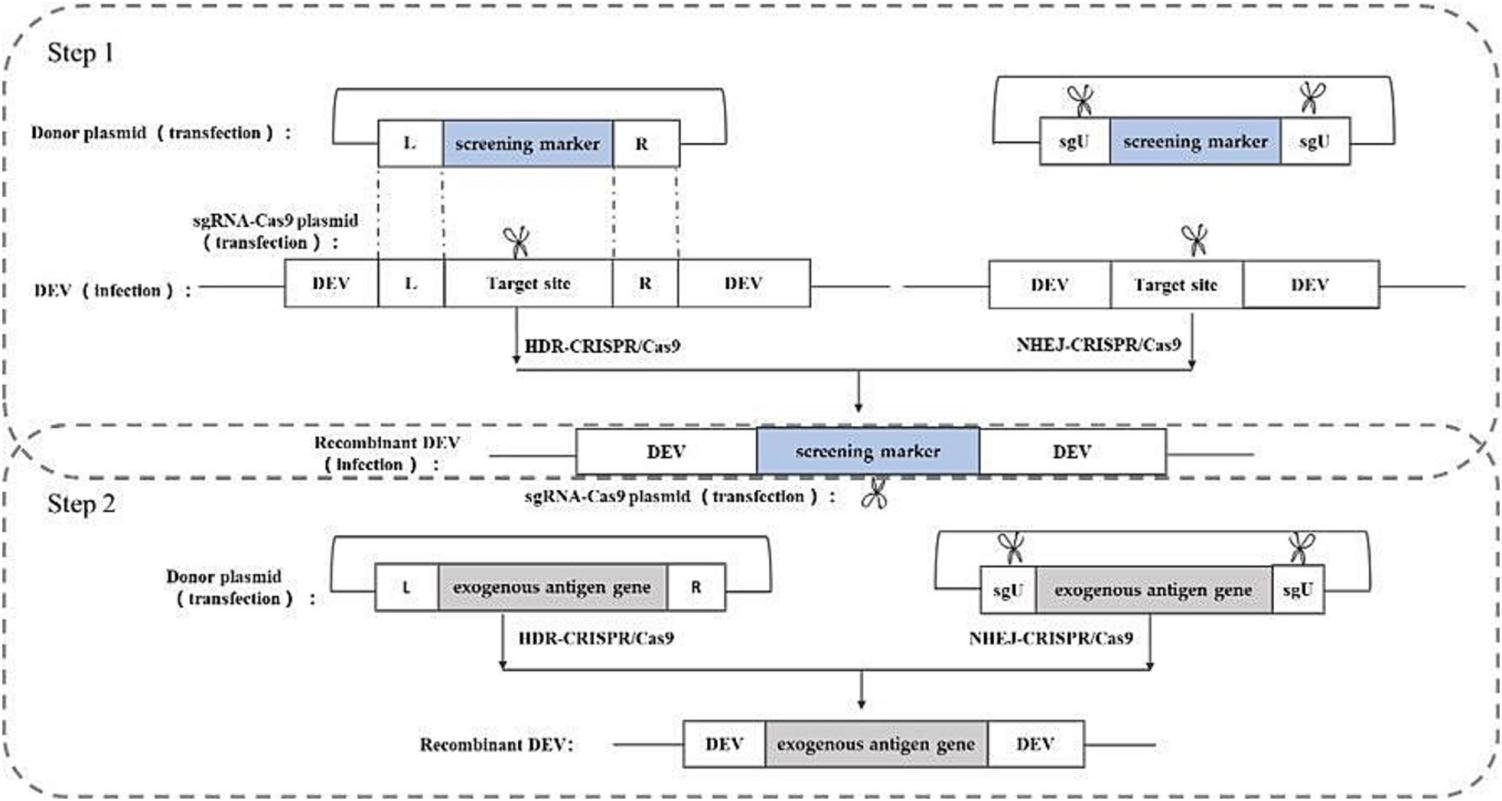
Recombinant DEV construction strategy based on CRISPR/Cas9 gene editing technology. sgU is a scrambled sequence with no homology to human, chicken, pig, prokaryotic, or viral genome sequences. L and R represent the left and right homologous arm sequences, respectively.

Studies have shown that based on these two repair methods, the construction of corresponding donor plasmids and appropriate screening methods can knock-in exogenous genes into the herpesvirus genome and construct recombinant herpesviruses expressing exogenous genes (34, 35). In the rescue of recombinant viruses, both repair-based exogenous gene knock-in strategies entail the construction of sgRNA-Cas9 plasmids targeting the cleavage site sequences and donor plasmids containing the exogenous genes. The HDR-based knock in strategy requires donor plasmids containing homology arm sequences on both sides of the insertion site, whereas the NHEJ-based knock-in strategy does not necessitate homology arm sequences on either side of the insertion site. However, the NHEJ-based knock-in strategy requires an additional sgRNA-Cas9 plasmid to linearize the donor plasmid. The construction strategy is illustrated in Figure 5.

Chang et al. (36) utilized the NHEJ-CRISPR/Cas9 coupled with the Cre-Lox System to insert the HA gene of H5N1 into the UL26/UL27 region of the DEV genome. Similarly, Apinda et al. (37) employed the same strategy to insert the ompH gene of *Pasteurella multocida* into the UL55/LORF11 and UL44/44.5 loci. This represents the first application of the CRISPR/Cas9 system to insert highly immunogenic genes from bacteria into the DEV genome, facilitating the rapid and efficient construction of a multivalent vector vaccine. This underscores the substantial potential of duck enteritis virus as a vector. While most current applications of CRISPR/Cas9 gene editing rely on NHEJ repair due to its high efficiency, HDR repair offers greater accuracy. Numerous successful cases of HDR repair-based CRISPR/Cas9 have been reported in constructing other herpesvirus vector vaccine (34, 38, 39).

The advantages of this method are the higher efficiency of rescuing recombinant viruses and the simplicity of the method, and the disadvantages are the presence of off-targeting, which may lead to unintended insertions or deletions.

Table 1 outlines the key points and the advantages and disadvantages of four methods for constructing recombinant DEV. Each method has its specific applications and limitations. The choice of method depends on the conditions of the laboratory and the specific requirements of the task. In practical applications, researchers need to weigh these pros and cons and select the method that best suits their research objectives.

**Table 1:** Advantages and disadvantages of four methods for constructing recombinant DEV.

Construction methods	Key point	Advantages	Disadvantages
HR	Recombination efficiency and screening of recombinant DEVs	The operation is simple and the cost is relatively low.	The rescue efficiency of recombinant DEV is relatively low.
BCA	Construction of DEV-BAC infectious clones	After obtaining DEV-BCA, the genetic modification of the DEV genome becomes more convenient, and the rescue efficiency of recombinant DEV is relatively high.	The construction of rDEV-BCA is both time-consuming and labor-intensive.
Fosmid	Determination of fosmid plasmid combinations	The process is efficient, and the resulting recombinant DEV does not require screening or purification.	It is necessary to identify a fosmid combination that can efficiently recombine into DEV.
CRISPR/Cas9	Selection of sgRNA and screening of recombinant DEV	The operation is straightforward, and the rescue efficiency of recombinant DEV is high.	Improper selection of sgRNA may lead to off-target occurrences, potentially causing unintended insertions or deletions of bases, which presents challenges for the later certification of the vaccine.

### 3.5 Strategy for the construction of polyvalent duck enteritis virus vectored vaccine

DEV serves as a highly promising viral vector capable of delivering antigenic genes from one or more pathogens, thereby constructing polyvalent vaccines that significantly broaden the scope of vaccine protection. Two main strategies exist for constructing polyvalent recombinant DEV vectored vaccines that are similar to those used to construct other herpesvirus vectored vaccines.

The first strategy employs a polycistron expression scheme, which utilizes elements such as an internal ribosome entry site (IRES) or a self-shearing polypeptide 2A (2A) to link multiple antigenic genes to form a eukaryotic expression cassette, which is then inserted into the DEV genome. Gergen et al. (40) have employed this strategy and successfully constructed vaccines expressing the Newcastle disease virus (NDV) fusion (F) gene and the infectious laryngotracheitis virus (ILTV) gD and gI genes in a recombinant turkey herpesvirus (HVT). This recombinant virus provided excellent protection against all three pathogens, ILTV, NDV, and Marek’s disease virus (MDV), after a single inoculation via the in-ovo or SC route.

Another strategy is to insert different antigenic genes into different loci of the DEV genome, respectively. Following this strategy, Zhao et al. (28) constructed a recombinant duck enteritis virus named rDEV-dH5/H7. The virus carried the hemagglutinin genes of two H5 viruses and one H7 virus. Animal studies showed that rDEV-dH5/H7 behaved similarly to its parental DEV viruses in inducing neutralizing antibody responses and protection against lethal DEV attacks. More importantly, rDEV-dH5/H7 was able to induce potent and long-lasting hemagglutinin-inhibiting antibodies against different H5 and H7 viruses, thus providing comprehensive protection against homologous and heterologous highly pathogenic H5 and H7 influenza virus attacks in ducks. In addition, this strategy was also used by Tang et al. to successfully generate a triple-inserted recombinant HVT-VP2-gDgI-HA vaccine by inserting the gD-gI gene of ILTV and the hemagglutinin expression cassette of the H9N2 AIV into different loci of the recombinant HVT-IBDV VP2 viral genome (41).

## 4 Advantages and limitations of DEV as a vaccine vector

### 4.1 Advantages of DEV as a vaccine vector

As a member of the *herpesviridae* family, DEV is considered an ideal live viral vector for the development of multivalent vaccines. DEV possesses a large genome that includes several non-essential regions for replication, such as US2, SORF3/US2, SORF3, US7/US8, UL2, UL27/UL26, UL41, and SORF11/UL55. The presence of these regions facilitates the insertion of foreign genes, thereby allowing for customized genetic engineering of the virus.

Within the host, DEV demonstrates rapid replication capabilities and can stimulate a comprehensive immune response within hours after vaccination, including humoral immunity and cellular immunity. It is noteworthy that the intensity and effectiveness of this immune response seem to be unaffected by the presence of maternal antibodies in the host. Furthermore, when used as a vaccine, DEV can establish persistent infection in the host, thereby providing long-term and stable immune protection. Zou et al. (42) inserted the (HA) gene of H5NI into the DEV vaccine strain C-KCE to obtain the recombinant virus C-KCE-HA. After a single immunization, Hemagglutination Inhibiting Antibodies were detected in the serum of immunized ducks, and the number of IFN *γ*-secreting cells in the splenocytes increased significantly.

In terms of safety, the natural host range of DEV is limited to ducks and does not pose a threat to other livestock or humans, which ensures the safety of its use as a live viral vector vaccine. Although recombinant DEV does not infect chickens, its transient replication in chickens is sufficient to express and present foreign antigenic proteins, effectively stimulating the host to produce an immune response. Based on these characteristics, DEV has been widely used in the development of vaccines against a variety of avian pathogens, including but not limited to avian influenza virus (28, 43), Newcastle disease viruses (NDV) (44), infectious bronchitis viruses (22), duck Tembusu viruses (24), Duck hepatitis A virus (29, 45), and even *Pasteurella multocida* (46), etc., providing an important means of disease prevention and control for the poultry industry. Table 2 details research data on various recombinant DEV vector vaccines that are critical to understanding the efficacy and potential applications of recombinant DEV vector vaccines harboring different pathogen antigen genes.

**Table 2:** Development and evaluation of recombinant DEV viral vector vaccines as immunogens for birds.

Pathogens	Exogenous genes	Insertion sites	Vector	Promoters	Construction methods	Immunization Dose/Route	Animal model	Efficacy	Reference
IBV	N’S or S1 gene	US10	Clone-03	CMV	HR	10^6.0^PFU/intramuscularly (i.m.)	4-week-old SPF chickens/10^6.0^ EID_50_ virulent IBV strain/oculonasal route(o.n.)	All recombinant DEV viruses showed significant protection against clinical diseases, with the rDEV-S vaccine providing better protection	(22)
H9N2 AIV	HA	UL2	attenuated DEV strain C20E85	HCMV-IE	HR	(1) 10^3^ TCID_50_/ (i.m.) (2) 10^2^ ~ 10^6^ TCID_50_ /(i.m.)	(1) 4-week-old SPF chickens /1000-fold minimum lethal dose of DEV strain AV1221/(i.m.) (2) 4-week-old SPF chickens/10^8^ EID_50_ H9N2 virus (A/duck/Guangdong/08) /intravenous injection (i.v.)	10^3^ TCID_50_ of rDEV-∆UL2-HA induced solid protection against lethal DEV challenge and completel prevented H9N2 AIV viral shedding	(23)
H5N1 AIV	HA	US2’gIgE	C-KCE	CMV	HR	5,000 TCID_50_/ (i.m.)	4-week-old ducks/10^7^ TCID_50_ lethal DEV AV1221 viruses	recombinant DEVs could induce efficient partial protection against a lethal DEV challenge, at a lower level than the parental DEV	(21)
	HA	UL55/LORF11	C-KCE	pMCMV	BAC	10^6^TCID_50_/ (i.m.)	5-weeks-old commercial ducks / 100 LD_50_ virulent DEV/ (i.m.)	The DEV vaccine provides 100% protection against duck plague	(25)
	HA	gB/UL26	C-KCE	Chicken *β*-actin	BAC	(1)10^5^ PFU/ subcutaneously (i.h.) (2) 10^5^ PFU/ (i.h.)	(1)1-month-old SPF ducks/ 100-fold DLD_50_ virulent DEV/ (i.m.) (2) 1-month-old SPF ducks / 100-fold DLD_50_ H5N1 virus/ (i.m.)	C-KCE-HA immunization provided 100% protection against homologous and heterologous HPAIV H5N1 and virulent DEV attacks	(42)
	HA	UL44	VAC	CMV	BAC	–	–	–	(55)
	HA	UL41’US7/US8	Vaccine strains	SV40	Fosmid	(1)10^5^ PFU/(i.m.) (2)10^5^ PFU/(i.m.)	(1)4-week-old SPF ducks /100-fold LD_50_ virulent DEV/ (i.m.) (2)4-week-old SPF ducks /100-fold LD_50_ H5N1virus/ intranasally (o.n.)	rDEV-us78HA provided full protection against lethal H5N1 within 3 days of vaccination, matching live DEV vaccines’ efficacy against lethal DEV. However, rDEV-UL41-HA showed slightly lower effectiveness against lethal DEV and H5N1 challenges compared to rDEV-US7/8-HA.	(50)
	HA	UL26/UL27	ATCC® VR-684™	EF	CRISPR/Cas9	–	–	–	(36)
H7N9’H5N1 AIV	HA	SORF3/US2’US7/US8’US8/US1	Vaccine strains	SV40	Fosmid	(1) 10^3^ ~ 10^5^ TCID_50_/ (i.m.) (2) 10^5^ TCID50/(i.m.)	(1) two-week-old SPF ducks/100 DLD_50_ virulent DEV/ (i.m.) (2) two-week-old SPF ducks/10^6^ EID_50_ AIV/(o.n.)	The rDEV-dH5/H7 vaccine virus induces an antibody response against DEV, H5 and H7 viruses in ducks, providing complete protection against lethal DEV and different H5 and H7 viruses.	(28)
NDV	F’HN	US7/US8	Vaccine strains	SV40	Fosmid	10^3^ ~ 10^5^ TCID_50_/ (i.m.)	two-week-old SPF chickens/10^3^CLD_50_ highly virulent NDV strain F48E9 / (i.m.)	rDEV-F induced 100% protection against a lethal NDV challenge in chickens with a minimum immune dose of 10^4^ TCID_50_, whereas rDEV-HN did not induce effective protective immunity in chickens.	(44)
DTMUV	E	SORF3/US2	C-KCE	hEF1α	BAC and Cre/ Loxp	10^5^ PFU/ (i.h.)	SPF ducks/100 DLD_50_ virulent DEV/(i.m.)	C-KCE-E induces antibody responses against DTMUV and is not significantly different from C-KCE in its protective efficacy against lethal DEV attacks	(56)
	E	US7/US8	C-KCE	CMV	BAC	–	–	–	(24)
	PrM’E gene truncator(TE)	US7/US8	Vaccine strains	SV40	Fosmid	10^6^ TCID_50_ / (i.h.)	two-week-old SPF ducks / 100-fold DID_50_ of the DTMUV/ (i.m.)	After inoculation with two doses of recombinant virus, rDEV-PrM/TE completely protected ducks against DTMUV attack, whereas rDEV-TE provided only partial protection	(57)
Serotype 1 and 3 DHAV	VP1	UL26/UL27	C-KCE	Chicken β-actin	BAC	10^5^ PFU/ (i.h.)	one-day-old SPF ducks /100-fold LD_50_ DHAV-1 or DHAV-3/ (i.m.)	rC-KCE-2VP1 triggered effective humoral and cellular immune responses against DHAV-1 and DHAV-3 with a protection efficiency of 100%.	(58)
Serotype 3 DHAV	P1-3C	UL26/UL27’US7/8	C-KCE	Pec	Fosmid	(1) 1000-fold ELD50 / (i.h.) (2) 1000-fold ELD50/ (i.h.)	(1)SPF ducks/ 100 ELD_50_ virulent DHAV-3 A3 strain/ (i.m.) (2) SPF ducks / 1,000 minimum lethal doses of the virulent DEV CSC strain/ (i.m.)	The rDEV-UL26/27-P13C vaccine provided 90 and 100% protection against DEV and DHAV-3 infections, and the rDEV-US7/8-P13C vaccine provided 70 and 100% protection against DEV and DHAV-3 infections.	(29)
Serotype 1 DHAV	VP0	UL41	C-KCE		Fosmid	–	–	–	(45)
*Pasteurella multocida*	ompH	UL55/LORF11’UL44/44.5	Jansen strain	MLV	CRISPR/Cas9	100 μg/(i.m.)	3.5 × 10^3^ CFU/mL/ virulent *P. multocida* strain X-73 / (o.n.)	rOmpH-duck plague combination vaccine was capable of effectively protecting ducks against artificial *Pasteurella multocida* infection	(59)

The “/” symbol represents the non-coding region between two genes.

### 4.2 Limitations of DEV as a vaccine vector

DEV serves as a promising vector for developing recombinant vaccines, offering numerous advantages, yet it is not without its limitations. Primarily replicating in *Anseriformes*, DEV vectors ensure a degree of safety while also restricting their applicability across diverse avian species and hosts. As a herpesvirus, DEV presents significant challenges in constructing reverse genetics systems due to its large genome. Rescuing recombinant DEV is notably complex and laborious compared to other commonly used viral vectors such as adenoviruses and Newcastle disease virus (47, 48). Genetic stability is another concern; recombinant viral vector vaccines may undergo genetic mutations or recombinations after multiple passages, potentially affecting the vaccine’s stability and efficacy. In addition, large-scale vaccine production requires specific cell lines or avian embryos. Most DEV strains, especially vaccine strains, can only be grown in homologous primary cell culture systems of avian origin, and the lack of commercial cell lines further complicates the production of vector vaccines (49).

## 5 Factors affecting the protective efficacy of recombinant DEV

### 5.1 Selection of vector virus strains

The selection of vector virus strains plays a crucial role in determining the effectiveness and safety of recombinant DEV. To ensure safety, most studies have used a weak strain of DEV, such as C-KCE or VAC, which minimizes the risk of adverse reactions associated with vaccination. These strains exhibit reduced virulence compared to wild-type DEV strains, thereby reducing the likelihood of causing disease in vaccinated animals.

### 5.2 Properties and expression of exogenous proteins

To ensure the normal expression of exogenous genes and maximize the expression efficiency of the inserted exogenous genes, according to the principle of eukaryotic gene expression regulation, the inserted exogenous genes must contain expression regulatory elements, so that they can exist independently in the genome of the virus, so most of the exogenous genes inserted in the current study are in the form of expression cassettes. Additionally, to trigger both cellular and humoral immunity, the inserted genes are typically full-length or partial sequences of key immunogenic proteins from other pathogens, such as the HA gene from AIV, the F and HN genes from NDV, the N and S genes from IBV, the E gene from DTMUV, and the ompH gene from *Bartonella multocida*.

However, some foreign genes exhibit low expression post-insertion, which can hinder immune response stimulation. This issue can be addressed through codon optimization, gene truncation, or the addition of signal peptides that enhance transcription and translation. For instance, Liu et al. (24) inserted various forms of the DTMUV E gene into the DEV genome, including origin E gene (E-ori), truncated E451-ori gene, codonoptimized E-dk gene optimized referring to duck’s codon bias, as well as the truncated E451-ch and E451-dk, Etpa-ori and Etpa-451-ori, which contain prefxing chick TPA signal peptide genes. Seven recombinant viral strains (rDEV-E) were constructed, and Western blot analysis revealed the presence of E or E451 proteins in infected CEFs, with the highest expression levels observed in CEFs infected with rDEV-E451-dk.

### 5.3 Insertion site

The site of exogenous gene insertion is another critical factor influencing the immune response elicited by recombinant duck enteritis virus. The DEV genome contains numerous replication non-essential regions suitable for exogenous gene insertion. Many studies have reported successful insertion and expression of exogenous genes in various regions of the DEV genome without compromising viral replication. These regions include US2, US10, US7/US8, UL26/UL27, UL44-44.5, UL41, UL55/LORF11, UL55-LORF11, and others, with a preference for non-coding regions between two open reading frames.

Multiple studies have evaluated the influence of different insertion sites on the immunogenicity and protective efficacy of recombinant DEV. For example, one study compared the immunogenicity and protective effects of two recombinant strains, with the HA gene of H5N1 inserted into the US2 and gIgE regions. The results showed no significant difference in antibody production or protection against DEV between the two strains, although both demonstrated lower efficacy than the parental DEV strain (21).

In another investigation, the HA gene was inserted into the UL41 and US7/US8 loci, yielding two recombinant viruses, rDEV-UL41HA and rDEV-US7/8HA. Both were found to be immunogenic and provided robust protection against a lethal DEV challenge. Importantly, rDEV-US7/8HA rapidly induced antibodies against the H5N1 virus and granted complete protection against the H5N1 virus challenge (50). Similarly, the DHAV-3 P1 and 3C genes were inserted into the non-coding regions between DEV’s UL26/UL27 and US7/8, resulting in the creation of rDEV-UL26/27-P13C and rDEV-US7/8-P13C. Both recombinant viruses triggered rapid immune responses and offered full protection against the DHAV-3 challenge. Furthermore, recombinant DEV expressing DHAV-3 antigens provided strong protection against lethal DEV challenge, with rDEV-UL26/27-P13C showing superior protection over rDEV-US7/8-P13C (29).

Although these studies suggest that different insertion sites may have some impact on the protective efficacy of recombinant DEV, the differences observed were not significant. However, the precise mechanisms underlying the effects of insertion sites on exogenous protein expression and the protective efficacy of recombinant viruses remain unclear. Therefore, further systematic studies are needed to elucidate these mechanisms and identify the optimal insertion sites for exogenous genes.

In Table 2, we have comprehensively summarized the relevant information on the recombinant DEV studied so far and their immune effects. Through the data presented, we can clearly observe the construction strategies of various recombinant DEV, such as the DEV strains used as vectors, the promoters, the insertion sites of foreign genes, the gene fragments utilized, as well as their immune effects in experimental animals or actual breeding environments. These data provide crucial reference information for further optimizing the development of recombinant duck enteritis virus vaccines.

## 6 Conclusion and outlook

The development of recombinant DEV vector vaccines not only provides a new strategy for the prevention and control of duck viral enteritis but also opens up new avenues for the integrated prevention and control of other poultry diseases. This new type of vaccine can achieve immune protection against multiple pathogens through a single vaccination, greatly simplifying the traditional immunization process, reducing the labor intensity and economic costs in the poultry farming industry, and alleviating the stress on animals caused by multiple vaccinations.

Looking back at the past three decades, the field of recombinant virus-vectored vaccines has undergone extensive research and evaluation. Yet, only a handful of cases have successfully transitioned to commercialization. Most of these commercialized vaccines are based on vectors such as the herpesvirus of turkeys and fowlpox virus, effectively addressing various poultry diseases including influenza (51), laryngotracheitis (52), Newcastle disease (53), and infectious bursal disease (54). Meanwhile, recombinant DEV vector vaccines, as rising stars, have rapidly become a research focus over the past decade. Researchers have leveraged cutting-edge biotechnologies such as Bacterial Artificial Chromosome, Fosmid rescue systems, and CRISPR/Cas9, significantly accelerating the development of recombinant DEV vaccine candidates, enhancing vaccine construction efficiency, and achieving greater precision and flexibility in vaccine design.

Even though recombinant DEV vaccines have demonstrated robust protective efficacy, their commercialization process lags behind that of other well-established viral vector vaccines, and there are no avian vaccine products based on this vector currently available on the market. Nevertheless, the research value of DEV as a highly promising vaccine vector cannot be overlooked. Future research should focus on optimizing insertion sites and promoters to enhance the expression efficiency and antigen presentation of foreign genes; delving into the correlation between the genetic stability and protective efficacy of recombinant DEV vaccines; analyzing the potential impact of foreign genes on the immunogenicity of the parental DEV virus; identifying the optimal balance between foreign gene expression levels and immune response capabilities; and ensuring the safety of recombinant DEV vaccines throughout production and administration et al.

Notably, studies on recombinant DEV vector vaccines for avian influenza prevention and control are particularly abundant (21, 23, 25, 50). Given that waterfowl serve as significant reservoirs and cross-regional transmission vectors for avian influenza viruses, their low immunization coverage poses a considerable challenge to avian influenza control. The avian influenza recombinant DEV vector live vaccine, with its advantage of “one shot, two diseases prevented, “rapid induction of protective effects, and long-lasting immunity, offers dual lifelong protection against avian influenza and duck virus enteritis in ducks and potentially a broader range of poultry species, representing a significant milestone toward the long-term control and potential eradication of avian influenza.

Looking ahead, the focus of scientific research should be on further optimizing the live recombinant DEV vector vaccine, ultimately achieving the industrialization and application of research achievements. This not only represents a pivotal breakthrough in the development of the poultry vaccine industry but also serves as a crucial safeguard for promoting the healthy and sustainable development of the poultry farming industry. This review, by outlining the construction methods of the recombinant live duck enteritis virus vector vaccine and the key factors influencing its immunological effects, aims to provide valuable references for researchers in related fields, working together to drive the in-depth development of research on the recombinant DEV vaccine.
